# Applying the layers of protection analysis (LOPA) method to high containment level biological facilities

**DOI:** 10.1038/s41598-025-88546-8

**Published:** 2025-02-05

**Authors:** Andrian Harsono, Ryan Waters, Jason Tearle, Graeme Harkess

**Affiliations:** The Pirbright Institute, Woking, GU24 0NF UK

**Keywords:** HAZOP, LOPA, Biocontainment, Safety Integrity Level, Safety instrumented function, Biosafety, Risk factors, Pathogens

## Abstract

The Layers of Protection Analysis (LOPA) method is a semi-quantitative risk assessment tool that is used to determine the ability of safeguards to protect against unplanned hazardous scenarios. One possible outcome of a LOPA is that existing and proposed safeguards are deemed sufficient to reduce the risk associated with the hazardous scenario to a level that can be deemed as acceptable. Alternatively, the LOPA may also show that the safeguards are insufficient and therefore additional Safety Instrumented Function(s) (SIF) would be required to reduce risk to an acceptable level. In the latter case, the LOPA method will inform the end user as to the reliability requirements of the safety function in question. The LOPA method has been used extensively in the process industries (e.g., oil and gas) as a useful tool to manage and understand risk and to demonstrate if the facility is ‘safe’ to operate, but much less so in the biosafety sector. This paper describes the LOPA method and provides some practical examples of how it may be applied in microbiological high Containment Level (CL) facilities.

## Introduction

International standards such as International Electrotechnical Commission’s (IEC) 61508 and 61511 have emerged as the standards for functional safety as applied to electrical, electronic, and electronically programmable systems. The requirement for compliance to these standards led to the development of the LOPA method. This method is still widely used in process industries (e.g., oil and gas) today to determine if new safety functions are required and how reliable they must be to provide a specified amount of risk reduction to achieve an acceptable level of risk. In 1997, attendees at the Center for Chemical Process Safety (CCPS) international conference and workshop on risk analysis decided that a book^1^ shall be written to describe the LOPA method. In addition to the book, academic papers have also been published for a closer study on this methodology^2–6^ and its variations.

This paper is split into sections that describe the basic steps required in carrying out a typical LOPA study:

1. Identify the hazardous scenarios to analyse and determine their target risk frequencies.
2. Identify all possible initiating causes/events that could result in the scenarios and estimate their frequencies.
3. For each initiating cause/event, determine all Independent Protection Layers (IPLs) and estimate their Probabilities of Failure on Demand (PFD).
4. Determine all applicable Conditional Modifiers (CMs) to the hazardous scenarios and to which initiating events they would apply to.
5. Calculate intermediate risk frequency and compare this with target risk frequency to see if additional layers of protection are required and how reliable they need to be.

A LOPA study can therefore be deemed to be successful when the below requirements are met:

- The hazardous scenario(s) identified is/are well defined and well understood by participants (i.e., little or no ambiguity).
- For each scenario, an appropriate target risk frequency is decided that is agreed and endorsed by the organisation.
- For each scenario, all initiating causes are identified, and their frequencies estimated to a level that is as accurate as available information allows.
- For each initiating cause, all IPLs are identified, and their PFDs estimated to a level that is as accurate as available information allows.
- All applicable CMs have scientific basis, are based on reasonable assumptions, and are correctly applied.
- Any residual risk is accurately calculated that provides an indication to any additional SIF required and how reliable they need to be.

This paper describes the application of this methodology in biological facilities to prevent the uncontrolled release of pathogens into the external environment that could result in a local or national outbreak. In the UK (where this paper is written), pathogens that affect humans and animals are regulated under specific legislation. For those that are pathogenic to people, the Advisory Committee on Dangerous Pathogens (ACDP) assesses biological agents on a scale from 1 (unlikely to cause disease) to 4 (causes severe disease) and produces the Approved List based upon this classification^7^. Consideration is also given to the likelihood of the spread to the community and the availability of treatment. Genetically modified biological agents do not appear in the Approved List. Animal pathogens are regulated under the Specified Animal Pathogens Order (SAPO), the purpose of which is to prevent the introduction and spread in the UK of these pathogens which if introduced could cause serious disease and economic loss. Under SAPO, animal pathogens may be classified on a scale from 1 to 4, the former being disease-producing organisms which are enzootic (native) and do not produce notifiable disease with the latter being disease-producing organisms which are either exotic or produce notifiable disease and have a high risk of spread from the laboratory^8^. An example of a human (ACDP4) agent would be a haemorrhagic fever virus such as Ebola or Marburg with an example of an animal (SAPO4) agent being the Foot and Mouth Disease Virus. There are corresponding CLs (CL4 being the highest) that laboratories must comply with, with more controls required as the level rises. At facility level, high containment facilities may appear similar although when handling human pathogens, further controls will be in place to prevent exposure of personnel to disease.

## Methodology

### Step 1: identify the hazardous scenario and determine its target risk frequency

Most LOPA studies are preceded by a Hazard and Operability (HAZOP) study, or a Structured What-If Technique (SWIFT) study. These studies help identify the possible hazardous scenarios that are reasonably foreseeable in any given process unit. Such studies also identify hazardous scenarios that are severe enough in consequence that further analysis (i.e., via the LOPA method) would be warranted. For high containment level microbiological facilities, such hazardous scenarios are typically events that result in an uncontrolled release of pathogens which could have a negative impact on either human health (e.g., human virus outbreak resulting in fatalities) or the environment by way of animal health (e.g., animal virus outbreak resulting in fatality of animals, resulting in significant impact to the local agricultural sector and the broader economy).

Examples of hazardous scenarios that may be selected for LOPA analysis:

- Loss of pressure cascade in the containment facility, resulting in potential transfer of aerosolised pathogens from a contained area to an uncontained area (i.e., in the “wrong direction”) or release of aerosolised pathogens into the external environment.
- Effluent Treatment Plant (ETP) discharging untreated effluent into drains or public sewers.
- Blocked drainage in the facility, resulting in contaminated water flowing back into the containment space and flooding the facility.
- Failed incinerator operation resulting in partial incineration of contaminated waste, where efforts to rectify the situation may result in a release of pathogens to the external environment.

Hazardous scenarios selected for a LOPA study carried out in the biosafety sector are expected to include those where there is a reasonably foreseeable chance of an unplanned release of pathogens that could result in infection of susceptible animals or humans in the facility or beyond. Inherent to this, is an understanding and definition of what constitutes a “release” of a pathogen. For example, a single virus particle released into the environment from a facility is unlikely to cause disease, therefore is unlikely to be considered as the “hazardous” event. For the purposes of LOPA studies which are semi-quantitative by nature, this paper recommends that “release” be quantified by infection probability which can be used as a CM. This is an acknowledgement that once an infection occurs, the organisation has limited control over the manner and speed with which the virus spreads, and so in this paper, “release” is not defined based on the geographical area covered by the outbreak or the number of animals the organisation thinks could be infected by the virus.

The probability of infection is dependent upon several factors as described below.

A characteristic that is commonly used to estimate the probability of infection is the Minimum Infective Dose (MID_50_), which is defined as the number of virus particles required to initiate infection in 50% of animals / humans exposed to it. The MID_50_ will vary between pathogen species, sub-strains within the pathogen species, or different susceptible species for the same pathogen. Even within the same susceptible species exposed to the same pathogen species, the MID_50_ will vary slightly depending on animal/human characteristics.

The route of infection is also considered when determining the severity of the hazardous event. For example, the risk of infection will change depending on whether the pathogen is vector-borne (e.g., mosquito, horsefly) as opposed to transmission via aerosol or fomite (e.g., door handles, handrails).

To summarise the above, the probability of infection and therefore potential severity of an uncontrolled “release” of pathogens is based on a combination of factors:

- The characteristics (e.g., MID_50_) of the pathogen in question (both animal and human). For example, some viruses are more infectious than others.
- Environmental factors, including:
  - The duration of the “release event”.
  - The number of susceptible humans and/or animals near the facility.
  - The route of infection (e.g., aerosol transmission, insects).
  - The stability of the pathogen with respect to outside temperature, humidity etc.

Once a hazardous scenario to be study is selected, a target risk frequency is selected. The target risk frequency (in yr^− 1^) for a hazardous scenario helps inform the level of risk that an organisation is willing to accept. HSE guidance^9^ has data about the fatality rate of workers in hazardous industries in the United Kingdom that is regarded as broadly acceptable risk (i.e., insignificant, and adequately controlled), and other fatality rates that the public may find tolerable “in the wider interest of society”. In LOPA studies, a target risk frequency of 1 in 100,000 years is commonly used for one worker fatality, and 1 in 1,000,000 years is commonly used for multiple fatalities. Note that these target frequencies will change depending on societal perceptions of risk.

At present, there is no equivalent data to suggest the risk levels of animal disease outbreaks that members of the public would be willing to tolerate “in the wider interest of society”. To analyse risks associated with animal pathogens via the LOPA method, organisations must therefore decide their own target risk frequencies associated with local (or national) outbreaks of animal diseases. In the example shown in Table 1, “Full sustained loss of containment & control at CL4, resulting in release of Specified Animal Pathogen Order level 4 (SAPO4) pathogen” is displayed as equivalent to the “Critical injury or work-attributable illness resulting in significant permanent disablement or death”, shown by their identical target risk frequencies. Organisations may choose different target risk frequencies based on the broader social, political, and economic impact of an animal disease outbreak in their respective countries. For example, countries that are heavily dependent on cattle farming for their economies are likely to be less tolerant towards the risk of an animal disease outbreak.

Table 1 Shows an example risk matrix with risk categories, consequence types, their descriptions, and example target risk frequencies. Ultimately, organisations must adopt a risk matrix appropriate to their own specific and unique circumstances. For LOPA studies analysing the loss of containment of human pathogens, the “Health and Safety” consequence type should be used since such an event would ultimately result in harm to human health.

**Table 1:** Example Risk Matrix. LOPA studies typically analyse scenarios of most severe consequence (bold).

	Consequence Type		
Risk Category	Health and Safety	Biohazard	Target Risk Frequency / yr^− 1^
Minor	Superficial injury causing temporary and slight discomfort but not requiring treatment	Reduced containment & control at CL2, but no potential for loss of containment.	10^− 1^
Moderate	Slight injury requiring first-aid treatment but no sickness absence	Containment & control at CL2 partially or temporary reduced such that a release could reasonably foreseeably result; **OR** reduced capability for containment & control at CL3, but not such that release could directly result	10^− 2^
Major	Significant injury or work- -attributable illness requiring treatment by a medical practitioner and up to 1 week sickness absence	Full sustained loss of containment & control at CL2, resulting in release of SAPO2 pathogen; **OR** containment & control at CL3 partially or temporary reduced such that a release could reasonably foreseeably result; **OR** reduced capability for containment & control at CL4, but not such that release could directly result.	10^− 3^
**Severe**	**Serious injury or work-attributable illness requiring hospitalisation of over 1 day, or substantive medical treatment for over 1 month, or otherwise resulting in over 1 week sickness absence**	**Full sustained loss of containment & control at CL3, resulting in release of SAPO3 pathogen;** **OR** **containment & control at CL4 partially or temporary reduced such that a release could reasonably foreseeably result.**	**10** ^**− 4**^
**Critical**	**Critical injury or work-attributable illness resulting in significant permanent disablement or death.**	**Full sustained loss of containment & control at CL4, resulting in release of SAPO4 pathogen.**	**10** ^**− 5**^

Step 1 is completed when the most appropriate consequence description is selected for the identified hazardous scenario, and its corresponding target risk frequency defined.

### Step 2: identify initiating events for the scenario and estimate their frequencies

LOPA studies require the identification of all possible initiating events that could result in the hazardous scenario that is to be assessed. Typical initiating events in microbiological facilities are not very different from other industries (e.g., oil and gas, pharmaceutical, etc.), but ventilation and electrical systems are of particular importance. This is because having robust directional airflow as a risk mitigation measure is specific to the microbiological, pharmaceutical, and medical sectors, and achieving this requires a reliable ventilation system which in turn requires a stable power supply. Some typical initiating events are:

- Blockage of filters and/or strainers.
- Failure of valves/dampers.
- Loss of power.
- Human error (e.g., operating the wrong valve, pressing the wrong button).
- Instrumentation or process control scheme failure.
- Pipe/flange/gland leaks.
- Failure of rotating equipment (e.g., fans, pumps, compressors, macerators).
- Natural disasters (e.g., large fires, floods, earthquakes) resulting in damage to large numbers of equipment on site simultaneously.

The frequencies (yr^− 1^) of these initiating events are generally defined by the facility given their experience with the above events. Some guidance on frequencies can also be obtained from other sources^10,11^.

Step 2 is completed once all possible initiating events and their frequencies are identified and recorded.

### Step 3: for each initiating event, determine all independent protection layers (IPLs) and estimate their probabilities of failure on demand (PFD)

For each initiating event that could lead to the hazardous scenario, IPLs help reduce the frequency of the scenario occurring. There are different types of IPLs available:

- **Active** (e.g., sensors that produce alarms upon detecting an unsafe situation, basic process control schemes).
- **Passive** (e.g., bunds, bursting discs, relief valves, blast walls).
- **Procedural** (e.g., personnel trained to respond in a certain way to unsafe situations, or to perform certain tasks before hazard is introduced to make the system safer, or to carry out regular maintenance to keep the equipment in good condition).

Implementing multiple IPLs of different types for the same initiating event will help to maximise the robustness of these protection layers. The IPLs must be:

- **Specific** for the prevention or mitigation of the consequences.
- **Independent** of other protection layers.
- **Dependable** in doing what it is designed to do.
- **Auditable** with regular validation, maintenance, and testing.

Some examples of IPLs include:

- Good engineering practice (e.g., equipment rated to the correct pressure and temperature with the correct material specification).
- Maintenance routines that are suitable for the equipment.
- Process control schemes.
- Alarms with well-defined operator responses.
- Pressure relief devices.
- Bunds.
- Deluge systems.
- Filters.

While PFDs are generally defined by the facility given their operating experience with the above events, guidance on these probabilities can also be obtained from other sources^11^.

Step 3 is completed once all possible IPLs to each initiating event and their PFDs are identified and recorded.

### Step 4: determine all applicable conditional modifiers to the hazardous scenario and to which initiating events they apply to

A CM is a factor that affects the probability of a consequence occurring after an initiating event. CMs may be events or conditions, but must be present, or happen, before the consequence is realised. CMs typically used in the process industry include:

- **Time at risk**: When the hazard is only present some of the time. This is very typical in batch manufacturing processes. This CM is best expressed as a percentage.
- **Occupancy**: When personnel are only present some of the time in the area where the hazardous scenario is expected to occur, typically for visual inspections, repairs, and maintenance. Once again, this CM is best expressed as a percentage.
- **Probability of ignition**: When the probability of ignition of a released flammable gas depends on the physical characteristics of the gas, density of electrical equipment in the area and whether said equipment is suitably rated for use in hazardous areas.

“Time at risk” and “Occupancy” are CMs that can also be used in the microbiological sector. However, instead of “Probability of ignition”, “Probability of infection” for animal pathogens or “Probability of fatality” for human pathogens should be used as explained in earlier sections of this paper. For the probability of fatality, the organisation may work with multiple pathogens, and therefore for purposes of the LOPA study, the “worst-case scenario” pathogen should be assumed, and given current scientific knowledge of that pathogen, the probability of fatality (i.e., mortality rate) of that pathogen be estimated and that number used as the CM.

As mentioned earlier, estimating the probability of infection for animal pathogens depends on the nature of pathogen and its release. Infection probabilities have been estimated in the case of burning pyres^12^, virus transmission via contaminated water^13^, and via aerosol transmission^14^. These can be used as templates for calculating or estimating infection probabilities, but organisations must take responsibility for ensuring that infection probability estimates are as accurate as can be given the specific pathogens that they work with.

### Step 5: calculate intermediate risk frequency and compare with target risk frequency

Each initiating cause (IC) has an associated event frequency. For example, an organization may decide that a pump can be expected to fail to perform its function approximately once every 10 years and that this failure could result in a hazardous scenario downstream. Specific to this event, there could be IPLs and CMs that apply to this scenario. Multiplying these numbers gives the “Mitigated Event Frequency” for this IC. These mitigated event frequencies are summed amongst all possible initiating causes to give the “Total Mitigated Event Frequency”, or “Intermediate Risk Frequency” as used in some industries. In the example given in Table 2, there are three different ICs, three different IPLs and two different CMs. A PFD is set to 1 if that IPL or CM does not apply to that IC and is therefore “guaranteed to fail”.

From the example below, the “Total mitigated event Frequency” is 2.6 × 10^− 4^ yr^− 1^ or one event every 3,846 years. If the organization has decided that the target risk frequency for this hazardous scenario should be say, one event every 100,000 years or 10^− 5^ yr^− 1^, then the calculated total mitigated event frequency is insufficiently low. In fact, the additional PFD required to achieve this target can easily be calculated as 10^− 5^ / 2.6 × 10^− 4^ = 3.85 × 10^− 2^. The organization may decide to introduce additional IPLs to further reduce this total mitigated event frequency or choose to install one SIF that has a PFD of 3.85 × 10^− 2^ or lower. A Safety Integrity Level (SIL) rating is typically allocated to the SIF depending on the resultant PFD that is required of it, which means this PFD corresponds to SIL 1:

**Table 2:** Example calculation of mitigated event frequency.

	Event Frequency (yr^− 1^)	IPL_1_ PFD	IPL_2_ PFD	IPL_3_ PFD	CM_1_ PFD	CM_2_ PFD	Mitigated Event Frequency (yr^− 1^)
IC_1_	0.1	0.2	0.1	1	0.1	0.2	4 × 10^− 5^
IC_2_	0.1	0.2	1	0.1	0.1	1	2 × 10^− 4^
IC_3_	0.01	0.2	0.1	0.1	1	1	2 × 10^− 5^
						Total =	2.6 × 10^− 4^

## Worked example: drainage blockage

With all the steps as described in previous sections, a worked example is shown in this section as illustrated in Table 3. Here, the hazardous scenario is the drainage backing up due to blockage(s) which could result in the building being flooded by contaminated effluent. This scenario is selected as an example since it is a realistic possibility for any microbiological facility with an effluent treatment plant and is a particularly hazardous scenario in the facility where the authors work.

**Table 3:** SIL rating vs. PFD or PFH. PFD is used in low demand mode (i.e., demand on SIF is expected to be less than once a year). PFH is used for high demand Mode (i.e., demand on the SIF is expected to be more frequent than once per year).

SIL Rating	Probability of Failure on Demand (PFD)	Probability of Failure per Hour (PFH)
1	10^− 1^ to 10^− 2^	10^− 5^ to 10^− 6^
2	10^− 2^ to 10^− 3^	10^− 6^ to 10^− 7^
3	10^− 3^ to 10^− 4^	10^− 7^ to 10^− 8^
4	10^− 4^ to 10^− 5^	10^− 8^ to 10^− 9^

In this example, the organization has selected a target risk frequency of 10^− 6^ yr^− 1^ (i.e., one in a million years). In this example, it has also been assumed that:

- Blockage in the main drainage header is observed in the drainage network once every decade.
- Operators closes a valve in error approximately once a year that would result in flooding.
- A control system failure occurs approximately once every decade that would result in flooding.
- A blockage in any one of the main drains is estimated to occur once every 5 years that would result in flooding.
- All IPLs have a PFD of one in ten (i.e., 0.1).
- The hazardous scenario is expected to place a demand on the SIF less frequently than once per year (i.e., Low Demand Mode).

After selecting all applicable IPLs and CMs, the mitigated event frequency is estimated to be 1.4 × 10^− 5^ yr^− 1^. This therefore means that the achieved risk frequency is not as low as that required by the organization, and that a new SIL 1 rated safety function (i.e., SIF) will be required to achieve the necessary risk reduction to achieve the acceptable level of risk i.e., the SIF will need to have a PFD between 0.01 and 0.1. In this example, the required PFD of the SIF is 7.14 × 10^− 2^.

It is important to emphasise that the objective of the worked example is only to demonstrate how to calculate the PFD of the resulting SIF. Ultimately, the initiating events, their frequencies, all IPLs and their associated PFDs will differ from one facility to the next and it is for organisations to estimate what they are.

## Limitations

Like all risk assessment methodologies, the LOPA methodology has its limitations. In the process industry, organisations assume failure rates of equipment and frequencies of other initiating events (e.g., human error). This principle also applies to the biology sector. Also, the probabilities of infection as mentioned in the references^12–14^ in this paper rely on many parameters such as environmental conditions (e.g., wind speed, temperature, humidity) and parameters specific to how susceptible certain humans and animals are to certain pathogens. It is therefore essential for LOPA study participants to appreciate that infection probabilities and other conditional modifiers will inherently contain uncertainties. To address this, it is recommended that LOPA studies consider the most realistic worst-case scenarios in all parameters used; this adds a level of conservatism to the numbers used. It is strongly recommended that all LOPA studies are conducted by experienced facilitators to ensure that these studies can produce meaningful outcomes for the organisation.

## Conclusion

The LOPA is but one of initial steps in a lifecycle of activities to ensure that high-containment facilities can be operated safely. The example as shown in Table 4 shows how the LOPA methodology allows an organisation to formally record possible hazardous scenarios and currently existing safeguards. In this example, the organisation has not sufficiently reduced risk to an acceptable level. The organisation will therefore need to take further action to ensure that risk is reduced further. The widely used international standards on functional safety (IEC 61508 / 61511) provide guidance on the subsequent phases of the safety lifecycle and describes how any installed SIFs must be appropriately specified, designed, installed, operated, maintained, and decommissioned.

**Table 4:** Complete worked example of a LOPA study.

SIF Description	None currently in place.						
Hazardous Scenario	Blockage found in drainage network. This leads to significant amount of untreated effluent backing up and spilling out all over the ground floor. If no action taken, contaminated water may leave the building and seep into the groundwater which may contaminate the water in the nearby meadow. If the water is consumed by susceptible animals, this could lead to an infection of a SAPO4 pathogen and may result in a national outbreak.						
Mode of operation	Low Demand (i.e., demand on SIF expected to be less than once a year).						
Hazard category	Biorisk - Animal Health Hazard	Target Risk Frequency (yr^− 1^) selected by organisation	1.00E-06				
		Total Mitigated Event Frequency (yr-1)	1.40E-05				
		PFD Target	7.14E-02				
		SIL Target	SIL 1				

No.	Initiating Cause/Event Description	Frequency (yr^− 1^)	IPLs and Conditional Modifiers - PFD				Mitigated event frequency (yr^− 1^)
			A	B	C	D	
1	Blockage in the drainage header due to gradual build-up of material. Experience shows that this will happen in the facility approximately once every decade.	0.1	0.1	0.1	0.1	0.01	1.00E-06
2	Operator closes valve in error. Experience shows that this could happen once a year.	1	0.1	0.1	0.1	0.01	1.00E-05
3	Control system failures that result in route to all downstream tanks being unavailable.	0.1	0.1	0.1	0.1	0.01	1.00E-06
4	Blockage in one of the many drains in the building. From experience, approximately once every 5 years.	0.2	0.1	0.1	0.1	0.01	2.00E-06
			Total Mitigated Event Frequency (yr^− 1^) =				1.40E-05

	IPL and conditional modifier Description	PFD					
A	Drainage level switches available in every drain to indicate that flooding is imminent.	0.1					
B	Valve position feedback available to notify control room if valves are in the wrong position.	0.1					
C	Washdown procedures are followed daily by competent personnel to clean away larger pieces of debris before washdown.	0.1					
D	Probability of SAPO infection as calculated by research paper published by institute XYZ.	0.01					

This paper suggests some initiating causes, independent protection layers and conditional modifiers that are specific to the biology sector. This paper aims to provide a useful guide to end-users as they carry out LOPA studies in their microbiological facilities. As this is a semi-quantitative technique, all numbers used here are estimates and organisations are responsible for ensuring that all estimates and assumptions are as accurate as can be for the most realistic worst-case scenarios. The LOPA methodology prompts organisations to think about the hazards and risks that are present in their facilities, and to consider if current safeguards are as reliable and effective as they think they are.

As scientists strive for ground-breaking research, it can be easy to lose sight not only of the risks that they face but also considerations as to whether these risks are adequately managed. With the right mentality, culture, and tools, the authors hope that the LOPA methodology will become an integral tool in reducing the frequency and impact of incidents involving unplanned release of pathogens from containment facilities.

## Data Availability

All data generated or analysed during this study are included in this published article.
